# Effects of sclerosing agents on head and neck hemangiomas: A systematic review

**DOI:** 10.4317/jced.56143

**Published:** 2019-11-01

**Authors:** Rúbia-Teodoro Stuepp, Fernanda-Marcello Scotti, Gilberto Melo, Etiene-de Andrade Munhoz, Filipe Modolo

**Affiliations:** 1 Postgraduate Program in Dentistry, Federal University of Santa Catarina (UFSC), Florianopolis, Brazil; 2 Department of Dentistry, Federal University of Santa Catarina (UFSC), Florianopolis, Brazil; 3 Department of Pathology, Federal University of Santa Catarina (UFSC), Florianopolis, Brazil

## Abstract

**Background:**

The aim of this study was to systematically review the literature for studies that investigated the effects of sclerosing agents on head and neck hemangiomas.

**Material and Methods:**

Clinical trials, cohort studies, and descriptive studies were considered eligible and selected in a two-phase process. Six main electronic databases, in addition to three grey literature databases, were searched. Risk of bias (RoB) was assessed using the “Meta-Analysis of Statistics Assessment and Review Instrument” checklist. From fifty-six considered eligible, five were finally included.

**Results:**

One article were judged at low, one at moderate, and three at high RoB. The sclerosing agents investigated were sodium tetradecyl sulphate (n=2), ethanolamine oleate (n=1), pingyangmycin (n=1) and bleomycin (n=1). Overall, good results were achieved on the treatment of head and neck hemangiomas with intralesional sclerotherapy. Most commonly reported adverse effects included pain, swelling, fever, necrosis, transient facial palsy, and anorexia.

**Conclusions:**

Considering the limited number of included studies, intralesional sclerotherapy on the management of HN hemangiomas presented overall good results with minor adverse reactions, especially in regards to smaller lesions.

** Key words:**Sclerotherapy, sclerosing solutions, vascular neoplasms, hemangioma.

## Introduction

Vascular anomalies comprise a heterogeneous group of lesions that have been classified by the International Society for the Study of Vascular Anomalies (ISSVA) into vascular malformations and vascular tumors (1). Hemangioma is a common type of benign vascular tumor that usually affects newborns and infants, although in some cases it might persist into adulthood (2). It should be mentioned that misdiagnosed of vascular tumors and vascular malformations is still recurrent and this can lead to misconduct, therefore, caution should be exercised regarding assessment of these conditions.

Approximately 10-12% of children at 1 year of age are affected by this condition (2) and nearly 60% of hemangiomas are located in the head and neck (HN) region, (3-5) specially the face, oral mucosa, lips, and tongue (2,4). Some subsets of hemangioma have been documented. Congenital hemangiomas (CH) are fully formed at birth and according to its clinical course, can be subdivided into two major subgroups: rapidly involuting congenital hemangioma (RICH) and noninvoluting congenital hemangioma (NICH) (6). The first one rapidly regress within 3 to 5 months and the second remains static in their clinical course (7).

Infantile hemangiomas (IH) are the most common benign vascular tumor of childhood and differs from CH in histologic features and immunophenotype, as well as clinical presentation and behavior (7). At birth, IH often appears as a precursor lesion, such as an area of telangiectasia or small purple area, then increases and become more recognizable. A fast growth occurs at three to five months; thereafter it usually involutes in several year, although abnormal texture, color, or residual fibroadipose tissue might persist on the overlying skin (3,8).

About 50% of hemangiomas have a complete resolution in 5 years and 70% in 7 years. Most of the lesions are small and usually no treatment is indicated (9). However, treatment is often indicated for those large lesions that present rapid growth, located in cosmetically areas, or presenting complications, such as ulceration, pain, bleeding, secondary infection, and tissue deformation (3,4,10). Possible psychosocial consequences on the affected child and family should also be considered with regard to treatment decisions (11).

To date, there is yet no consensus on the treatment of hemangiomas. Nonetheless, surgical excision is no longer the first choice (4) since it is related with complications such as bleeding, scarring, organ and tissue dysfunction (12), nerve damage (13), and often results in residual pathology (5). Still, many nonsurgical treatments have been attempted, including systemic propranolol and corticosteroids, interferon-α, lasertherapy, embolization, cryotherapy, radiotherapy, intralesional sclerotherapy, among others (10). Sclerosing agents applied intralesionally promotes lesion reduction and sclerosis (13). However, a wide range of sclerosing agents have been documented and its effectiveness and safety remains unclear (13).

The aim of this systematic review (SR) was to investigate the effectiveness and safety of sclerosing agents on HN hemangiomas and provide to physicians and dental clinicians an evidence-based therapeutic decision-making.

## Material and Methods

A SR protocol was registered at Prospective Register of Systematic Reviews under the registration number CRD 42018100394 (14). The reporting of this SR was based on PRISMA recommendations (15). The acronym PICOS (Population, Intervention, Comparison, Outcome, Studies) was used to formulate the question of this study, of which: P) individuals diagnosed with HN hemangiomas; I) intralesional sclerotherapy; C) other therapies, placebo, or pre-treatment status; O) lesion size reduction and complications; and S) clinical trials, cohort studies, or case-series with at least 10 participants. Only articles published in Latin Roman alphabet were considered.

The following exclusion criteria were applied: 1) studies that evaluated animals; 2) case-series with less than 10 participants with HN hemangiomas; 3) studies that combined sclerosing agents with other therapies; 4) studies investigating sclerosing therapy for vascular malformations or that did not provide separate data for hemangiomas; 5) studies investigating sclerosing therapy for peri- or intra-orbital vascular tumors; 6) studies in which outcomes for different sclerosing agents or different lesion sites were not reported separately; 7) abstracts, reviews, case-reports, protocols, laboratory research; 8) studies not published in the Latin Roman alphabet; and 9) full-text not available.

Search strategies were elaborated for the following electronic databases: Embase, Latin American and Caribbean Health Sciences (LILACS), PubMed, SCOPUS, The Cochrane Library, and Web of Science. In addition, a grey literature search was conducted on Google Scholar, Open Grey, and ProQuest. All searches were performed from the starting coverage date through May 12, 2019. Detailed search strategies are provided in  Table 1,  Table 1 continue.

**Table 1:**
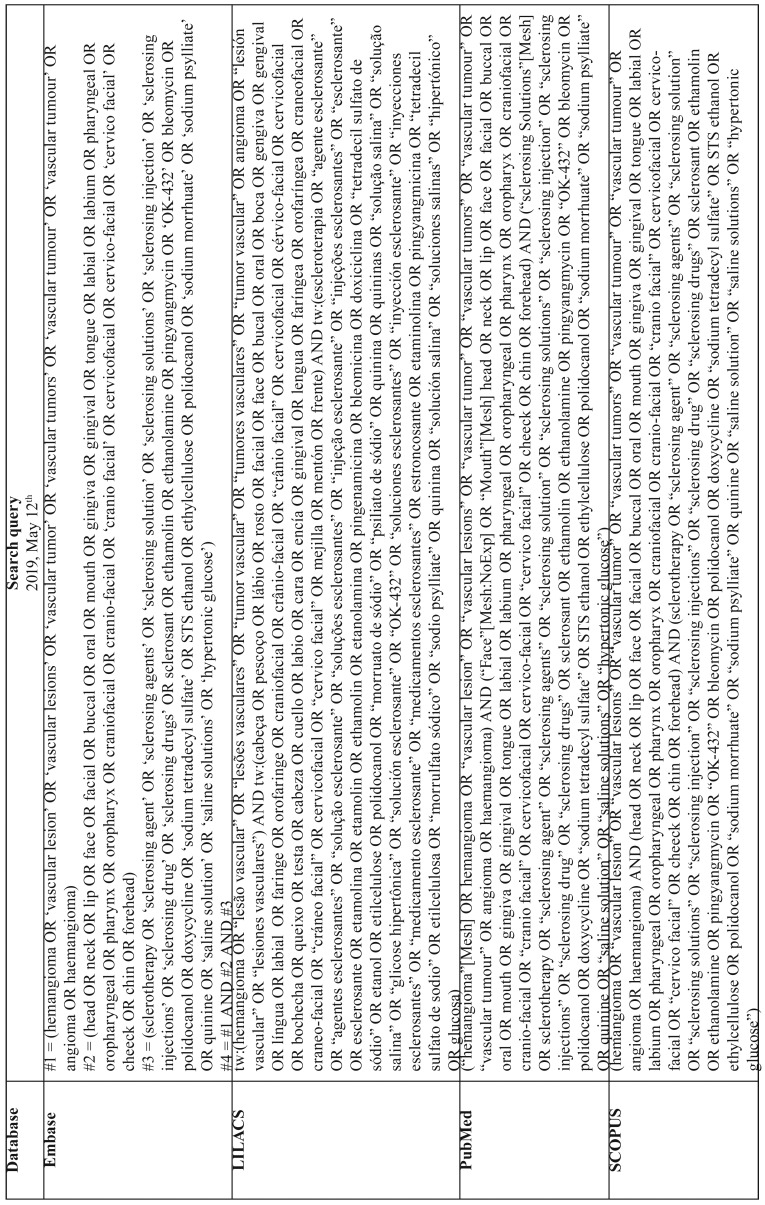
Data search strategy.

**Table 1 continue:**
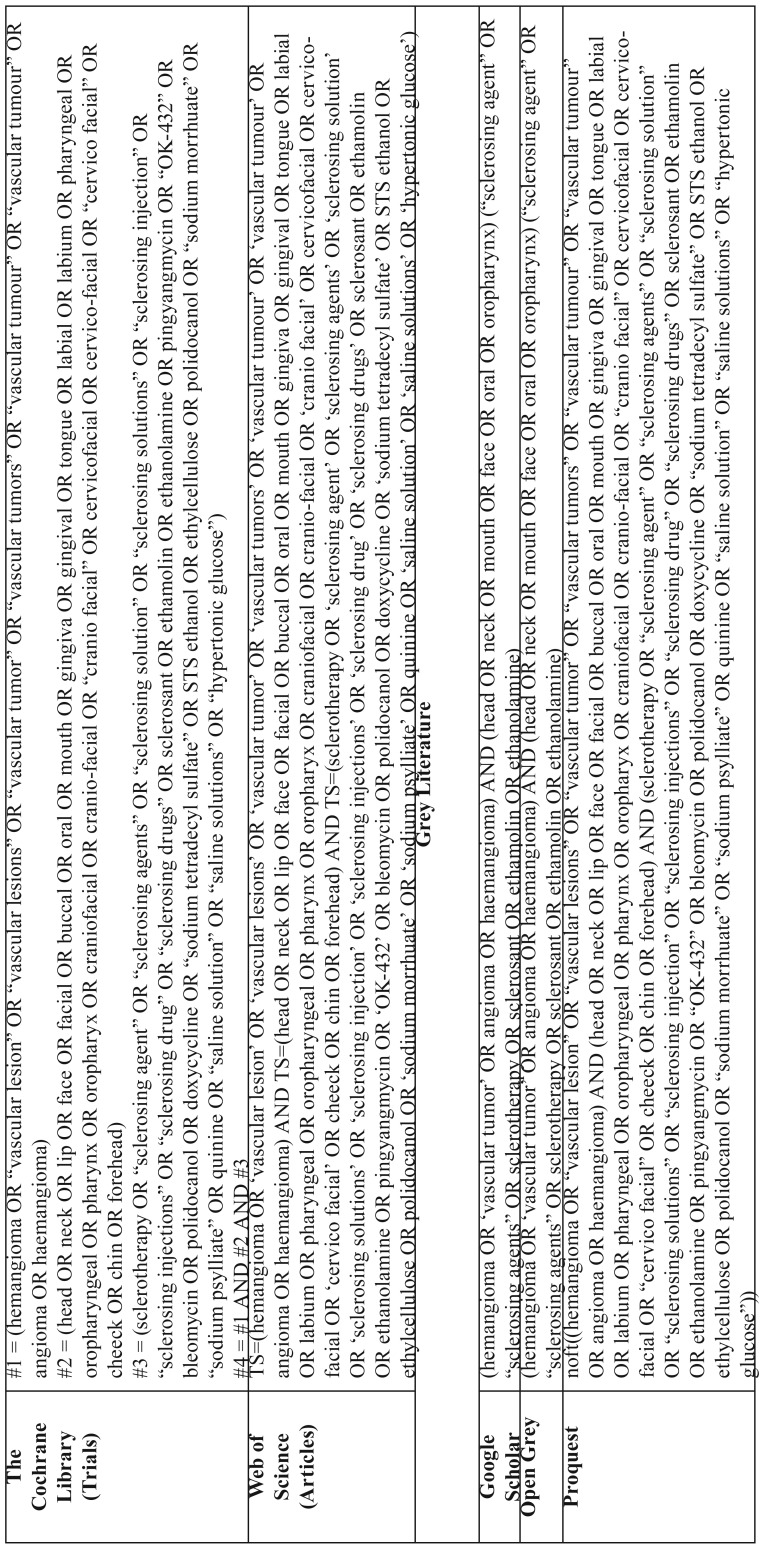
Data search strategy.

Furthermore, following the recommendation by Greenhalgh and Peacock (16), reference lists of included studies were hand-searched to assess relevant references. Reference management and removal of duplicates were performed using software (EndNote X7, Thomson Reuters).

The selection process was performed in two phases. Phase-1 was carried out in a web application (Rayyan®, Qatar Computing Research Institute). Firstly, two blinded reviewers (R.T.S and F.M.S.) screened title and abstracts of all identified studies and discrepancies were resolved by a consensus discussion; if necessary, a third reviewer was involved (G.M.). Thereafter, the same reviewers applied the eligibility criteria to full-text articles; if a consensus was not achieved, the third reviewer was consulted. Studies were included for analysis if minimum inclusion criteria were met.

Two blinded reviewers (R.T.S. and F.M.S.) collected data from included studies; information was then crosschecked to warrant integrity of contents. Gathered data consisted of study characteristics, population characteristics, intervention characteristics, outcome measures, and adjustment factors.

Risk of bias (RoB) was independently assessed by two reviewers (R.T.S. and F.M.S.) using the Joanna Briggs Institute Meta-Analysis of Statistics Assessment and Review Instrument (MAStARI), specific for descriptive studies. A third reviewer (G.M.) was involved in case of disagreements. Studies were categorized as “high” when reaching up to 49% score “yes”; “moderate” when reaching 50% to 69% score “yes”; and “low” when the study reached more than 70% score “yes”.

Lesion size reduction and occurrence of collateral effects related to sclerotherapy (e.g. pain, ulceration, fever, hypo or hyperpigmentation, and others) were evaluated by means of absolute or relative differences between baseline and follow-up evaluations. In order to standardize results, values were described in percentage.

## Results

**Figure 1:**
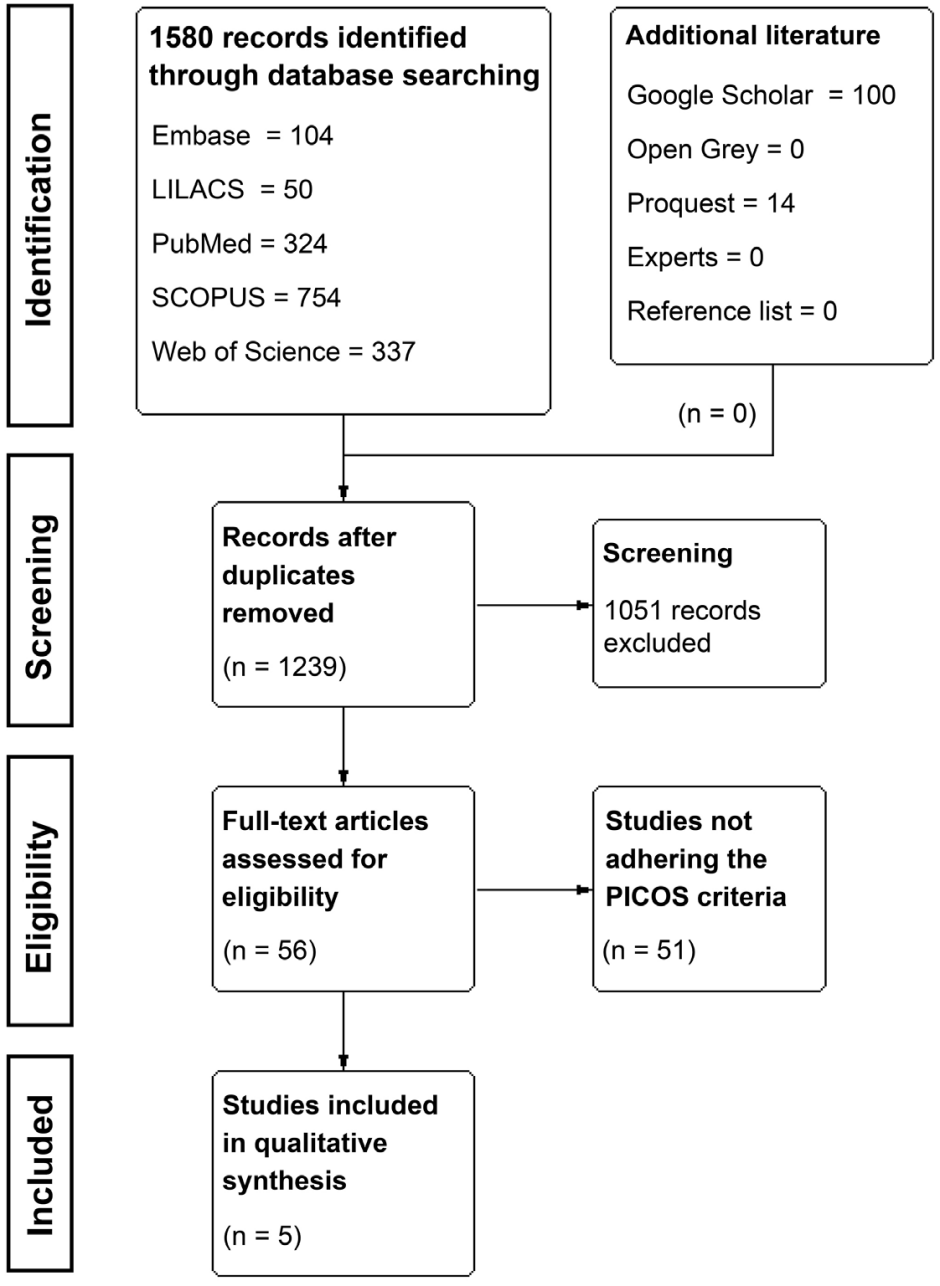
Flow diagram of literature search and selection criteria (adapted from Preferred Reporting Items for Systematic Reviews and Meta-Analysis and generated using the software Review Manager 5.3, The Cochrane Collaboration).

The search strategy, after removing duplicates, resulted in 1239 records. Following title and abstract screening, fifty-six articles were considered eligible for full-text reading, of which 51 were excluded with reasons accordantly to eligibility criteria ( Table 2,  Table 2 continue). Thereafter, five studies were included for qualitative analysis (Fig. 1).

**Table 2:**
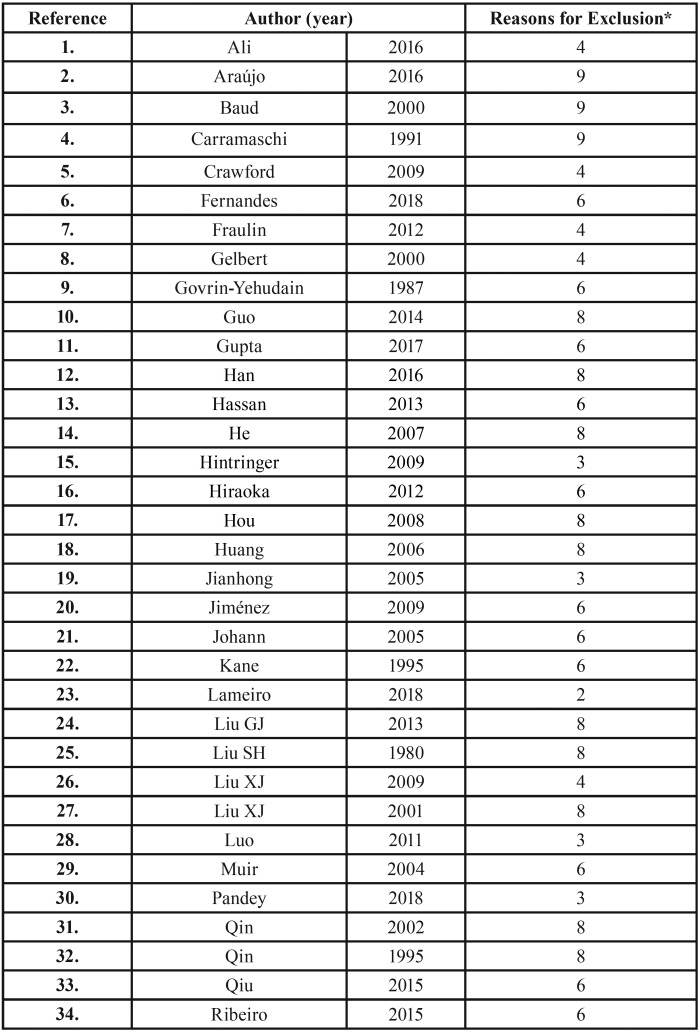
Articles excluded and the reasons for exclusion (n=51).

**Table 2 continue:**
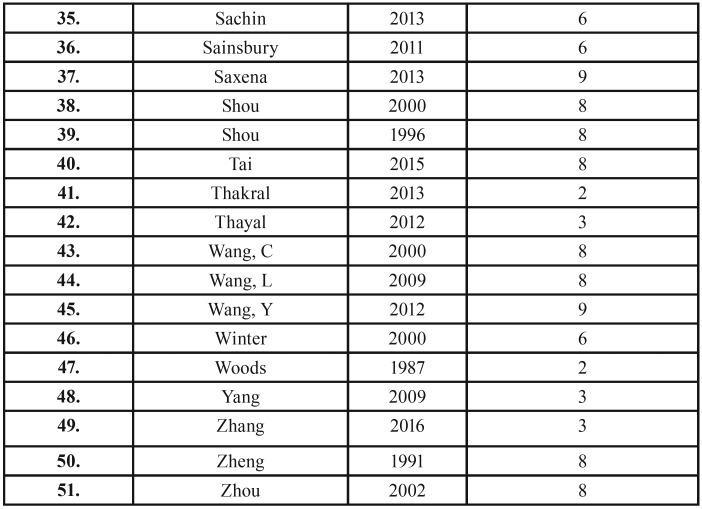
Articles excluded and the reasons for exclusion (n=51).

All included articles were descriptive studies, of which 4 were prospective case-series (5,17-19) and 1 a retrospective study (20). One study was conducted in China (5), one in India (17), one in Brazil (20), one in South Africa (19), and 1 in Israel (18). Most studies were written in English (5,17-19) and 1 in Portuguese (20). Sample size ranged from 13 (20) to 66 (5) participants. Participants’ age ranged from 2 months (5) to 79 years (18). Only 1 study did not provide any information considering adverse reactions (20). More information about study characteristics is available in  Table 3,  Table 3 continue,  Table 3 continue-1.

**Table 3:**
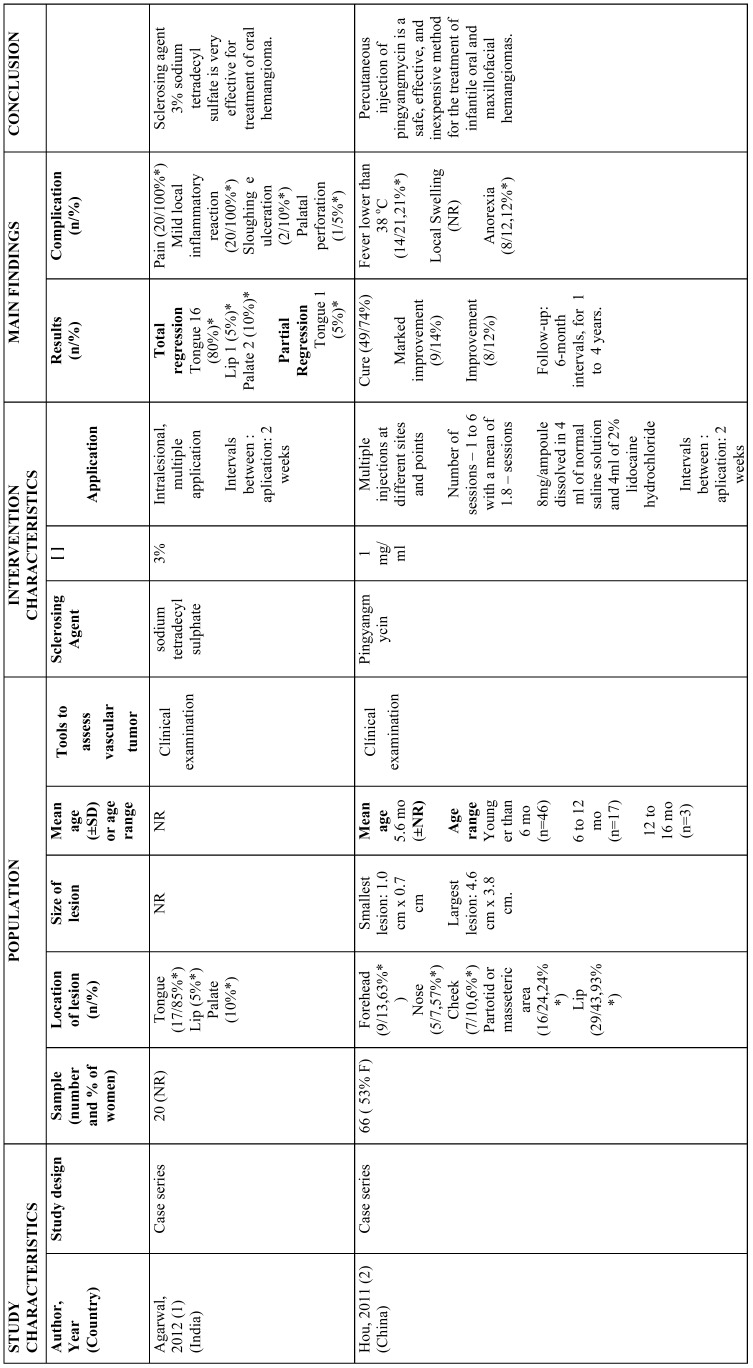
References.

**Table 3 continue:**
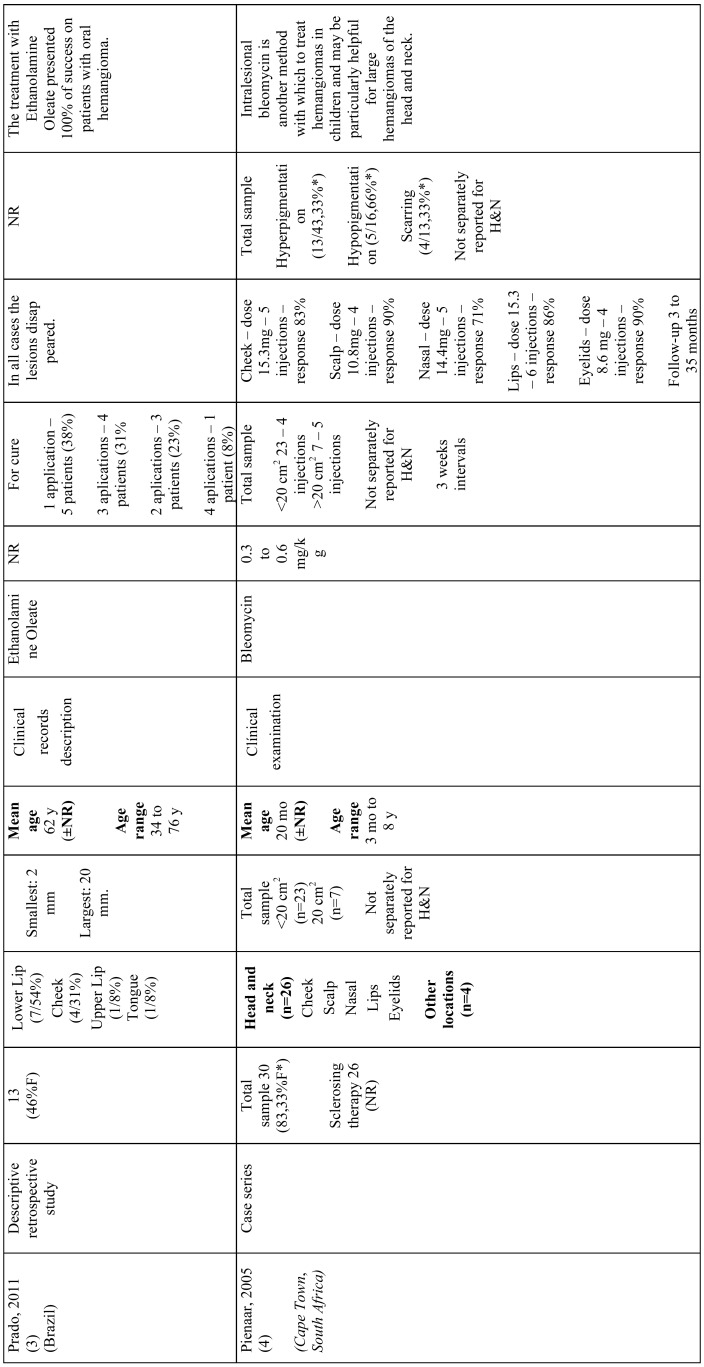
References.

**Table 3 continue-1:**
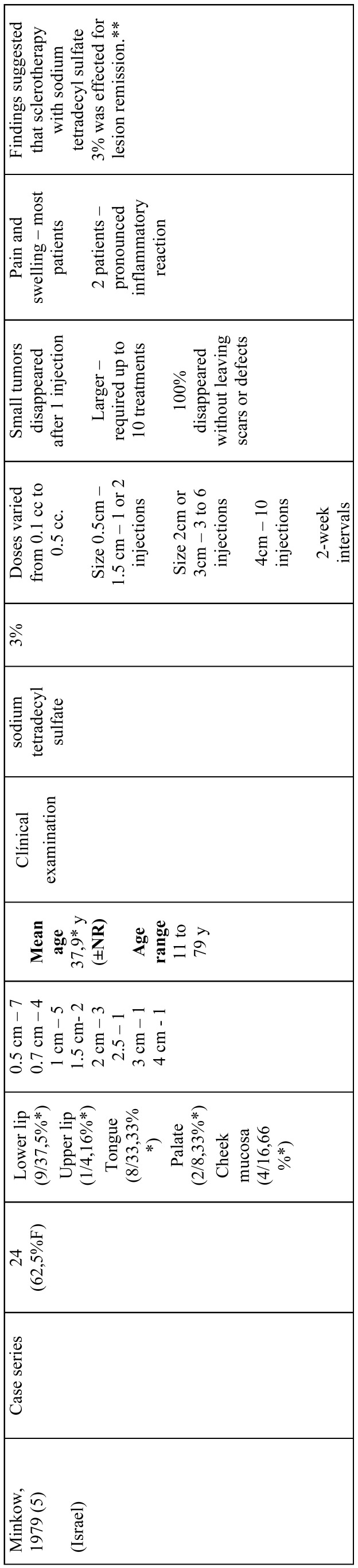
References.

**Figure 2:**
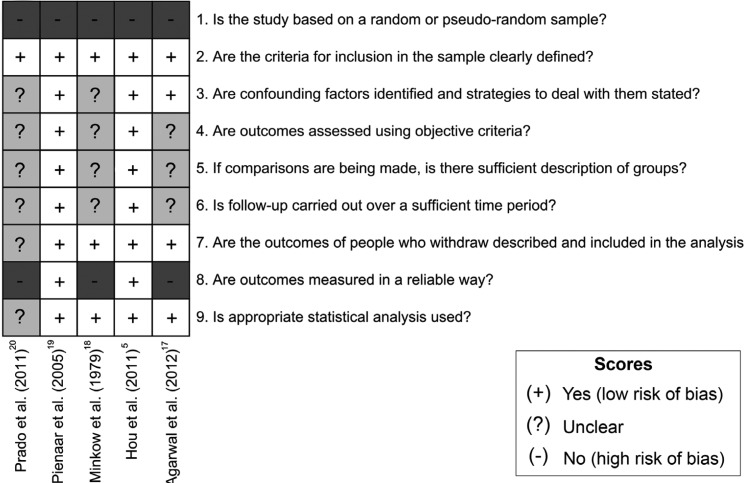
Risk of bias summary, assessed by the Joanna Briggs Institute Meta-Analysis of Statistics Assessment and Review Instrument (MAStARI): author’s judgments for each included study (generated using the software Review Manager 5.3, The Cochrane Collaboration).

Overall, one study were judged at low, one at moderate, and 3 at high RoB. No included study investigated randomized or pseudo-random populations; however, this was expected since participants must present benign HN hemangiomas. Regarding confounders, three studies provided detailed description of methods (5,17,19), whilst 2 did not provide sufficient information to permit a clear judgment (18,20). Moreover, two studies used objective outcome measures, whilst others used subjective measures (17,18,20). It should be noted that only 2 studies carried out an appropriate follow-up (5,19). More information regarding RoB assessment is available in Figure 2.

-Results of individual studies

Agarwal *et al.* (2011) (17) investigated the effect of intralesional STS 3% on hemangiomas of the tongue, lip, and palate in 20 participants (mean age not reported). Total regression was observed in 19 (95%) individuals, whilst partial regression was observed in a single case (5%). Pain and mild local inflammatory reaction were observed in all cases, sloughing and ulceration in 2 (10%), and palatal perforation in 1 case (5%).

Minkow *et al.* (1979) (18) studied the effect of STS 3% on hemangiomas located in the lips, tongue, palate, and cheek mucosa in 24 participants (mean age 37.9 years). Overall, total remission was observed after a single application in small tumors (0-1.5 cm), whilst larger tumors (2-4 cm) required from 2 to 10 applications. No scars or defects were observed after treatment, however, most individuals (frequency not reported) presented pain and swelling and 2 (8.3%) had pronounced inflammatory reaction.

Prado *et al.* (2011) (20) evaluated the therapeutic effect of intralesional EO (concentration not specified) on generic reported hemangiomas located in the lips, cheek, and tongue in 13 participants (mean age 62 years). Total regression was observed in all cases, although number of applications differed. A single application was required in 5 (38.5%) participants, whilst 3 (23.1%) required 2 applications, four (30.8%) required 3 applications, and in 1 individual (7.7%), four applications were required. Side effects were not reported in this study.

Pienaar *et al.* (2006) (19) assessed the effect of intralesional bleomycin (0.3 to 0.6 mg/kg) on hemangiomas in 30 individuals (mean age 20 months). From these, twenty-six (86.7%) individuals had lesions located in the cheeks, scalp, nose, lips, or eyelids. All individuals received 4 to 6 applications. Response-rates (rates higher than 90% were considered complete involution) regarding HN lesions were as follows: cheeks (83%), scalp (90%), nose (71%), lips (86%), and eyelids (90%). It should be noted that adverse reactions were not separately reported for HN lesions and that 4 (13.3%) individuals had lesions in other anatomical sites. Nevertheless, hyperpigmentation was observed in 13 (43.3%) individuals, hypopigmentation in 5 (16.7%), and scarring in 4 (13.3%).

Hou *et al.* (2011) (5) investigated the effectiveness of intralesional pingyangmycin (1 mg/ml) on infantile hemangiomas of the forehead, nose, cheek, parotid/masseteric area, and lip in 66 individuals (mean age 5.6 months). The number of applications ranged from 1 to 6 and follow-up time from 1 to 4 years, occurring every 6 months. Complete cure of lesions was observed in 49 (74%) individuals, whilst 9 (14%) had substantial improvements, and 8 (12%) showed mild improvements. Adverse reactions observed were local swelling (frequency not reported), fever lower than 38oC in individuals 14 (21.2%), and anorexia in 8 (12.1%).

-Synthesis of results 

Clinical and methodological heterogeneity across studies were considered high. Since few studies were included and different substances and therapeutic protocols were observed, statistical pooling of data using meta-analysis was not considered appropriate.

Considering different sclerosing agents observed, two studies investigated STS 3% (17,18), one EO (20), one assessed bleomycin A5 hydrochloride in the concentration of 0.3 to 0.6 mg/kg (19), and 1 study evaluated pingyangmycin (1 mg/ml) (5).

Most studies presented good results for sclerotherapy regarding lesion reduction. Total remission in all participants was reported by Prado *et al.* (2011) (20) and Minkow *et al.* (1979) (18), whilst total remission or major improvements were reported by Agarwal *et al.* (2011) (17), Hou *et al.* (2011) (5), and Pienaar *et al.* (2006) (19). The number of applications varied according to the size of lesion.

The following side-effects were reported across studies: pain (17,18); swelling (5,18); sloughing, ulceration, and palatal perforation (17); fever (5); anorexia (5); hyper/hypopigmentation (19); scarring (19); and local inflammation (17,18). The follow-up times were considerably discrepant, with a minimum follow-up of three months (19) and maximum of four years (5).

## Discussion

Sclerotherapy on the management of hemangiomas has been a topic of interest in several studies as they are considered a conservative method and are usually well tolerated. Still, the literature is sparse considering its effectiveness. This SR was performed to synthesize data gathered and guide clinical conducts and further researches regarding this topic. It was observed that, considering lesion remission, intralesional sclerotherapy presented overall good results with minor side effects. Nonetheless, evidence was considered weak and further controlled trials and objective outcome measurements are recommended.

In this SR, the studies of Agarwal *et al.* (2011) (17) and Minkow *et al.* (1979) (18) investigated STS 3%, of which total remission was observed in all (18) or in the majority of participants (17). STS is detergent sclerosant that produce endothelial damage through multiple mechanisms leading to thrombosis and fibrosis (21,22). This agent is considered to be less aggressive than absolute ethanol and has been used to treat HN vascular lesions (21).

Commonly reported adverse reactions in included studies investigating STS 3% were pain, swelling, local inflammatory reaction, sloughing, ulceration and palatal perforation (17,18). These findings are in accordance with current literature since swelling, pain, discoloration of the lesion, ulcer (23), and also fever and rashes (24) are common adverse reactions observed. The most reported side effect is tissue hyperpigmentation, although it is probably associated with use of inappropriately high concentrations or unexpectedly fragile veins. It should be highlighted that tissue necrosis has also been reported after administration of recommended doses, nonetheless, use of more dilute preparations of STS can lead to comparable results with decreased risk of necrosis (22).

Furthermore, a single included study (20) evaluated intralesional sclerotherapy with EO and, although evidence was considered weak, total remission of the lesions were observed in all participants. EO is a detergent agent (21) that acts as sclerosant through endothelium damage, leading to thrombosis and fibrosis (12,25). The effectiveness of EO is proposed to be similar to STS and safer compared to ethanol (21). No adverse effects were reported in the study of Prado *et al.* (2011) (20).

Still, since data were collected from charts, gaps in information about treatment outcomes might be present. Nonetheless, most commonly reported adverse effects of EO in current literature are redness, inflammation, pain during injection, tissue necrosis, and anaphylaxis (25). Although the study of da Silva *et al.* (2014) (12) was excluded due to insufficient number of participants, EO was used to treat oral hemangiomas and only a local burning sensation was reported as a side effect during administration.

Regarding other sclerosing agents, one study investigated the effects of bleomycin A5 hydrochloride (19). Bleomycin is a chemotherapeutic agent and, because of its thrombogenicity capability, it has also been used in sclerotherapy (21). In the study of Pienaar *et al.* (2006) (19) bleomycin was used to treat hemangiomas. This agent showed good response-rates, including in lesions larger than 20 cm.

The collateral effects reported were hyperpigmentation, hypopigmentation, and scarring. Known collateral effects caused by bleomycin A5 are edema, ulceration, scarring, nausea, lack of appetite (26) and tissue atrophy (27). Soft tissue atrophy is partly attributed to necrosis, although it might affect normal cells as well, and becomes more evident as the child grow up, since the tissue fails to grow (27). This collateral effect may be related to high dose or concentration of the sclerosing agent (27).

A single included study (5) investigated Pingyangmycin to treat IH with maximum size of 4.6 cm x 3.8 cm, showing good results and few adverse reactions. Pyngyangmycin has a chemical structure similar to bleomycin, acting by damaging endothelial cells, which leads to collapse, shrinkage, and fibrosis of target tissues (27,28).

There are many available options to treat HN hemangiomas, including surgery, systemic drugs (e.g. propanolol, atenolol, steroids), laser (4,28), topical drugs (e.g. imiquimod), ultrasound (28), as well as sclerotherapy or just a follow-up (21), still there is not a gold standard. In cases of IH that are life-threatening, such as lesions obstructing the airway, systemic propranolol is considered the first choice of treatment (29). This method is also indicated in cases of existing or imminent functional impairment, ulceration, pain, and bleeding, as well as the risk of long-term or permanent disfigurement (30).

Propanolol is non-cardioselective blocker of beta-adrener¬gic receptors. It is proposed that propranolol inhibits vasodilation via beta-receptors, which decreases blood flow to the lesion; blocks the release of proangiogenic factors (e.g., VEGF, bFGF, MMP-2, and MMP-9), thus limiting the growth of IH; and induces apoptosis in endothelial cells, favoring tumor remission (30).

 Although propranolol is considered a conservative treatment, risk of systemic effects should be highlighted. The most common non-serious events related to oral propranolol are sleep disturbances, diarrhea, and constipation. Moreover, serious complications may occur, such as hypoglycemia or related seizure, bradycardia, hypotension, atrioventricular disturbances, and bronchospasm/ bronchial hyperreactivity (29). Due to potential systemic effects, topical administration has been suggested. A SR showed that good responses in size reduction with minimal side effects (itching and erythema) can be achieved, although treatment success might be related to longer treatment durations (31).

In this SR, primary studies were considerably heterogeneous, especially regarding methods. Since different sclerosing agents were used, no direct comparison could be performed. Moreover, results should be interpreted with caution, as the magnitude of observed effects might be overestimated due to lack of control groups. Therefore, athough overall good results were observed, further clinical studies with controlled design, standardized methods, and objective outcome measurements are recommended to better explore this topic.

Within the limitations of this SR, intralesional sclerotherapy with STS 3%, EO, bleomycin, and pyngyamicin on the management of HN hemangiomas presented overall good results with minor adverse reactions, especially in regards to smaller lesions.
